# Activity patterns of the nectar-feeding bat *Leptonycteris yerbabuenae* on the Baja California Peninsula, Mexico

**DOI:** 10.1093/jmammal/gyae092

**Published:** 2024-08-19

**Authors:** A Nayelli Rivera-Villanueva, Winifred F Frick, Tina L Cheng, Veronica Zamora-Gutierrez

**Affiliations:** Centro Interdisciplinario de Investigación para el Desarrollo Integral Regional (CIIDIR) Unidad Durango, Instituto Politécnico Nacional, 34220 Durango, Mexico; Laboratorio de Biología de la Conservación y Desarrollo Sostenible, Facultad de Ciencias Biológicas, Universidad Autónoma de Nuevo León, 66455 Nuevo León, Mexico; Bat Conservation International, 500 North Capital of Texas Highway, Austin, TX 78746, United States; Department of Ecology and Evolutionary Biology, University of California, Santa Cruz, CA 95064, United States; Bat Conservation International, 500 North Capital of Texas Highway, Austin, TX 78746, United States; School of Biological Sciences, University of Southampton, Highfield Campus, Southampton SO17 1BJ, United Kingdom; CONAHCYT-Centro Interdisciplinario de Investigación para el Desarrollo Integral Regional (CIIDIR) Unidad Durango, Instituto Politécnico Nacional, 34220 Durango, Mexico

**Keywords:** activity budgets, food availability, lesser-longed nosed bat, Mexico, nectar-feeding bats, reproductive condition, condición reproductiva, disponibilidad de alimento, México, murciélago magueyero menor, murciélagos nectarívoros, patrones de actividad

## Abstract

Temporal activity patterns of animals can indicate how individuals respond to changing conditions. Gregarious roosting bats provide an opportunity to compare activity patterns among individuals living in the same location to investigate how reproductive status or sex may influence activity budgets. We examined how the activity patterns of the nectarivorous bat *Leptonycteris yerbabuenae* vary depending on reproductive conditions, sex, and environmental conditions. We analyzed 5 years of individual mark-resighting data using daily detections of *L. yerbabuenae* marked with passive integrated transponder tags (PIT-tags) at 3 subterranean roosts on the Baja California Peninsula, Mexico. We derived 4 metrics using PIT-tag detections at roost entrances to calculate periods inside the roost and time spent outside the roost (time of emergence, returns to the roost, hours inside the roost, and hours of activity). We found differences among pregnant, lactating, and nonreproductive females for roost returns, hours inside the roost, and hours of activity outside the roost. Lactating females spent the longest time outside the roost, suggesting that the energetic demands of lactation require longer foraging bouts. Contrary to our expectations, lactating females had the fewest returns to the roost during the night, suggesting that lactating females did not shorten foraging bouts to return to nurse pups. Activity patterns differed between females and males and among seasons associated with different food availability. Females had fewer returns during the night and spent more time outside the roost than males. The time of emergence for males was earlier than for females except during the nectar season when most females are reproductively active. Differences in activity patterns among reproductive status, sex, and environmental conditions show how individuals modify behaviors to meet their energetic demands. We demonstrate how mark-resighting data from PIT-tag systems at roost entrances can be used to compare activity patterns of gregarious roosting bats.

## Teaser

*Leptonycteris yerbabuenae* is a nectar-feeding bat and a colonially roosting species. Here, we assessed activity patterns among individuals to investigate how reproductive status, sex, and environmental conditions influence activity budgets.

## Abstract (es)

Los patrones temporales de actividad de los animales pueden indicar cómo responden los individuos a condiciones cambiantes. Los murciélagos que forman agregaciones en refugios brindan la oportunidad de comparar patrones de actividad entre individuos que viven en el mismo sitio, con el fin de investigar cómo influye el estado reproductivo o el sexo sobre dichos patrones. En este estudio, examinamos cómo los patrones de actividad del murciélago nectarívoro *Leptonycteris yerbabuenae* varían según su condición reproductora, su sexo y según las condiciones ambientales. Analizamos cinco años de datos de recapturas de individuos marcados mediante Transpondedores Integrados Pasivos (PIT-tags), utilizando detecciones diarias de *L. yerbabuenae* en tres refugios subterráneos en la Península de Baja California, México. Desarrollamos cuatro métricas a partir de las detecciones diarias en las entradas de los refugios, con la finalidad de delimitar los períodos que los individuos pasan dentro y fuera del refugio (hora de emergencia, frecuencia de regreso al refugio, horas de descanso y horas de actividad). Encontramos diferencias entre hembras preñadas, lactantes y no reproductivas en cuanto a la frecuencia de regreso al refugio, las horas dentro del refugio y las horas de actividad fuera del refugio. Las hembras lactantes pasaron más tiempo fuera del refugio, lo que sugiere que las demandas energéticas de la lactancia requieren períodos de búsqueda de alimento más prolongados. En contra de lo esperado, las hembras lactantes mostraron la menor cantidad de retornos al refugio durante la noche, lo que sugiere que las hembras lactantes no reducen los períodos de búsqueda de alimento para regresar a amamantar a las crías. Los patrones de actividad fueron diferentes entre hembras y machos, así como entre las temporadas con diferente disponibilidad de alimento. Las hembras regresaron menos durante la noche y pasaron más tiempo fuera del refugio en comparación con los machos. La hora de emergencia de los machos fue más temprana que la de las hembras, excepto durante la temporada de disponibilidad de néctar, que es cuando la mayoría de las hembras se encuentran reproductivamente activas. Las diferencias en los patrones de actividad en función del estado reproductivo, el sexo y las condiciones ambientales muestran cómo los individuos modifican su comportamiento para satisfacer sus demandas energéticas. Demostramos que los datos de recaptura de individuos utilizando los sistemas de marcaje PIT-tag en la entrada de los refugios se pueden utilizar para comparar los patrones de actividad de murciélagos gregarios.

Energy is the main limiting factor for wild animals as resources can fluctuate in space and time. Therefore, energy allocation is one of the main strategies that animals apply to optimize energy use and refers to the distribution of energy for growth, maintenance, storage, and reproduction (Perrin and Sibly 1993). Energy allocation in animals can be analyzed using different approaches such as somatic growth (Burril et al. 2018), survival rate (Post and Parkinson 2001), metabolic expenditure (Weathers and Sullivan 1993; Cunha et al. 2007), and through activity patterns (Burril et al. 2018). Animals have a differential energy allocation to efficiently obtain and use their energy for storage, maintenance, growth, and reproduction. In mammals, the main factors that affect activity patterns include reproductive stage and sex as well as environmental factors (i.e., temperature, humidity, precipitation, and food availability; Logan and Sanson 2003; Halle and Stenseth 2012; Monterroso et al. 2013).

Bats live near a negative energetic balance due to the high energetic demands required to sustain flight (Thomas and Suthers 1972; Voigt et al. 2011). Specifically, nectarivorous bats have a marginal gain of energy from their food sources compared to other trophic guilds, since pollen and nectar mostly contain carbohydrates and minimal amounts of protein (Voigt and Speakman 2007; Göttlinger et al. 2019). Thus, nectarivorous bats—compared to other trophic guilds—need to constantly search for food to compensate for the low energetic gain obtained from it (Kelm et al. 2011). Activity patterns of bats are highly influenced by food availability, sex, and reproductive condition (Barclay 1989; Wilkinson and Barclay 1997; Cryan et al. 2000; Dietz and Kalko 2007; Lučan and Radil 2010).

Female bats have higher energetic demands than males, especially when they are reproductively active, and they tend to compensate for these higher demands by adjusting their foraging behavior (Bronson 1985). Among female reproductive cycles, lactation is the most demanding physiological stage due to the increased energetic requirements of protein and calcium needed to produce milk (Speakman 2008). It has been found that the females of the nectarivorous migratory bat *Leptonycteris yerbabuenae* have a mixed reproductive strategy, where pregnant females act as capital-income breeders meaning that they accumulate nutrient reserves during their migration that later they use, together with the input of external nutrients for the development of offspring; while lactating females are mainly income breeders using external nutrients to subsidize milk production (Ramírez Hernández and Herrera 2016). The most energetically demanding period for males is during spermatogenesis and mating season, but even during mating season, males have lower energetic requirements than reproductive females (Pfeiffer and Mayer 2013). As an example of how energetic demands relate to activity patterns, Dietz and Kalko (2007) found that males have shorter bouts of foraging activity than females.

Environmental conditions can also influence activity patterns in bats (Swift 1980; Frick et al. 2012; Appel et al. 2019). The most relevant environmental variables influencing the activity patterns of insectivorous and frugivorous bats include food availability, temperature, precipitation, and moonlight (Appel et al. 2019). Among these, food availability is the most important since bats have to adapt their foraging activity based on the spatio-temporal distribution of food to obtain all the energy necessary for their survival (Barclay 1991; de Souza Aguiar and Marinho-Filho 2004; Scott 2004; Lang et al. 2006; Bogan et al. 2017). Precipitation, temperature, and moonlight can have a contrasting influence depending on habitat type, latitude, and species (Erickson and West 2002; Thies et al. 2006; Appel et al. 2019).

Despite the ecological and economic importance of pollinating bats (Kunz et al. 2011; Tremlett et al. 2021), few studies have investigated their activity patterns. We compared activity patterns of *L. yerbabuenae* to assess how reproductive condition, sex, and environmental factors may influence allocation of energy for foraging and roosting behaviors. We first ask how activity patterns (measured as time of emergence, frequency of returns to the roost, hours spent inside the roost, and hours of activity outside the roost) of females vary by their reproductive condition (nonreproductive, pregnant, and lactating). We predicted that lactating females would have the earliest emergence times and spend the most time foraging outside the roost to meet their high energetic demands, but would also return to the roost most frequently between foraging bouts to care for and nurse nonvolant pups. We predicted that pregnant females would stay the longest inside the roost and spend less time foraging outside the roost because of reduced maneuverability during pregnancy. We also asked how activity patterns differed by sex and environmental conditions. We predicted that females would emerge earlier and have longer hours of activity outside the roost than males, especially during the low food availability season.

## Materials and methods

### Study site

**Fig. 1.**
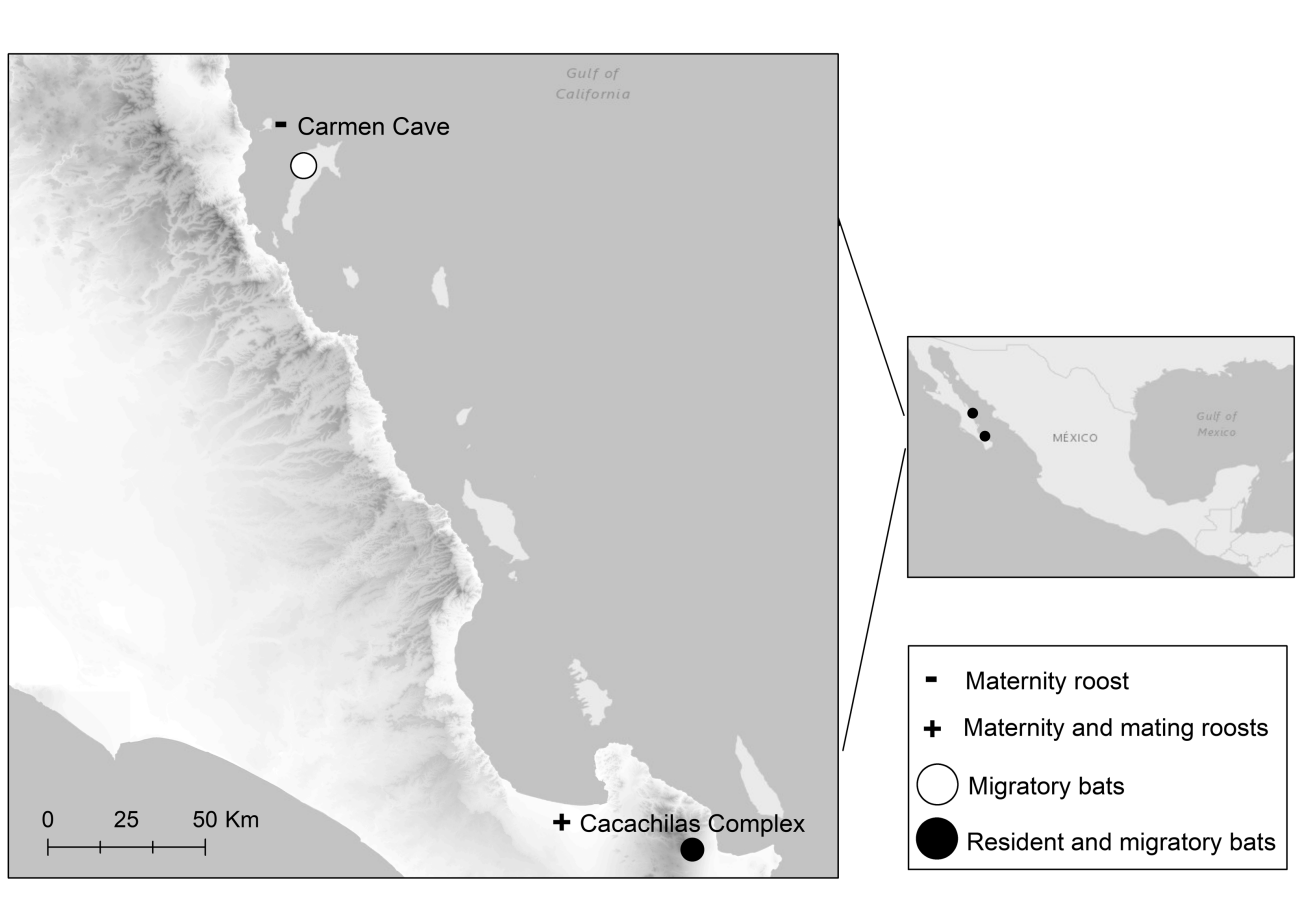
Study sites located in Baja California Sur, Mexico. Carmen Cave is a maternity roost with female *Leptonycteris yerbabuenae* bats and is seasonally occupied from April to July. The Cacachilas Complex includes a maternity and a mating roost with near year-round occupancy and includes both male and female *L. yerbabuenae* bats (Frick et al. 2018).

We studied the activity patterns of *L. yerbabuenae* at 3 subterranean roosts in Baja California Sur, Mexico (Fig. 1). These roosts have been monitored since 2015 using passive integrated transponder tags (PIT-tags) with Biomark IS1001 radiofrequency identification (RFID) transceivers attached to 15-m flexible cord antennae (Biomark, Inc., Boise, Idaho) installed at roost entrances (Frick et al. 2018). To examine whether activity patterns of females differed by their reproductive condition, we used the PIT-tag data obtained from 2013 to 2018 at a maternity cave located on Carmen Island (Carmen Cave). This roost is occupied only from late March through mid-July by reproductively active females that typically give birth in mid-April (Frick et al. 2018). To assess how activity patterns differ by sex and environmental factors, we used data from 1 maternity and 1 mating roost located in close proximity to each other (<1 km) in the Sierra de las Cacachilas. Both roosts host females and males throughout the year. Because these 2 roosts are located <1 km from each other and bats switch between the 2 roosts on a nightly basis (Frick et al. 2018), we treated these 2 roosts as a single site, and refer to these as the “Cacachilas Complex” (Fig. 1). Here, bats have been tagged from 2015 to 2018 across different seasons as there are resident and migratory bats present all year-round. Chivato roost is a predominately male roost with approximately 10 to 1,000 individuals, while Gitana is a maternity roost usually with fewer bats than Chivato at peak occupancy (Frick et al. 2018).

### Bat capture and handling

We captured bats using harp traps at roost entrances or entering a site and using hoop nets. We determined sex, age, and reproductive condition and measured mass and forearm length of captured individuals (Racey 2009). We marked 1,193 bats with PIT-tags by subdermally inserting a 12-mm tag premounted into a sterilized needle loaded in an applicator gun (Biomark, Inc.) under the dorsal skin (Kunz and Weise 2009) (Table 1). The insertion site was sealed using a fast-acting medical adhesive (3M Vetbond Tissue Adhesive). To ensure that pregnant females of Carmen Cave continued in that stage for the estimation of the metrics of activity, we excluded individuals who were in the late period of their gestation by gently palpating the abdomen to recognize prominent distension according to Frick et al. (2018). All captures followed the guidelines of the American Society of Mammalogists (Sikes et al. 2016).

**Table 1.** Total number of *Leptonycteris yerbabuenae* bats tagged and analyzed after applying the selection criteria at each site. The tagging occasions from Carmen Cave were from 2013 to 2018 for females only, and Cacachilas Complex were from 2015 to 2018. For Carmen Cave, only female bats were analyzed and based on their reproductive condition; while at Cacachilas Complex, bats were analyzed by sex.

Site	Reproductive condition/sex	Number of bats tagged	Number of bats analyzed
Carmen Cave	Lactating	183	92
	Nonreproductive	160	19
	Pregnant	113	38
	Postlactating	155	0
	Total	611	149
Cacachilas Complex	Females	333	112
	Males	249	185
	Total	582	297

### Metrics of activity

The Biomark IS1001 RFID readers with cord antennae were installed in roost entrances to allow continuous monitoring of bats as they enter and exit roosts without the need for physical recapture after tagging (Frick et al. 2018; van Harten et al. 2019, 2021). We excluded all detections in the first 3 days after a bat was tagged to avoid registering any abnormal behavior resulting from the stress of handling. To address our objective on how activity patterns are affected by the reproductive condition in female bats, we analyzed only the data for Carmen Cave. After discarding data of the 3 first days after tagging each bat, we selected the detections of only the next 14 following days to correctly assign reproductive conditions. To test for the influence of sex and environmental conditions, we analyzed the data from the Cacachilas Complex and used all detections from 2015 to 2019 for both males and females that followed our criteria of selection. In total, we analyzed tag detections of 149 of 611 tagged bats at Carmen Cave (19 nonreproductive, 92 lactating, and 38 pregnant females), and 297 of 582 tagged bats at Cacachilas Complex (185 males and 112 females).

**Fig. 2.**
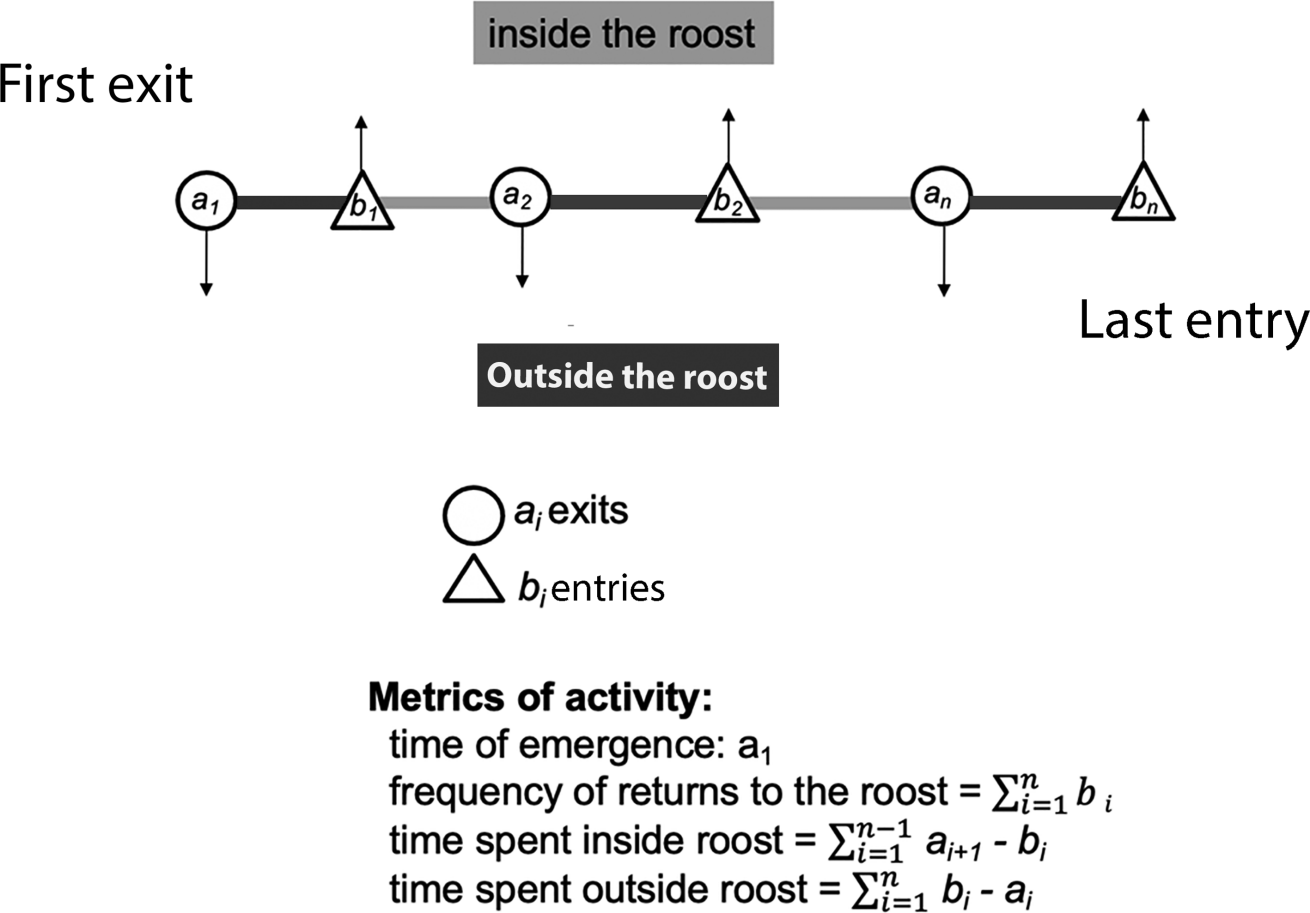
Schematic representation of the 4 metrics of activity calculated from PIT-tag detections recorded at 3 subterranean roost entrances. Detections were coded as exits and entries to the roosts. Detections in circle (*a*_*i*_) are considered exits and those in triangle (*b*_*i*_) are considered entries.

After data cleaning, we derived 4 metrics following certain rules to reflect the activity patterns of *L. yerbabuenae* from PIT-tag detections: roost emergence time; frequency of nightly returns to the roost; amount of time spent inside the roost per night; and time spent outside the roost per night (Supplementary Data SD1; Fig. 2). The definitions of each metric for activity patterns are as follows.

*Time of emergence*—the time of the first exit from the roost each night. For the Cacachilas Complex, we converted time of emergence to minutes before or after the local sunset time to normalize the exit time across seasons. For Carmen Cave, all data analyzed corresponded to the same season; thus, it was not necessary to convert time of emergence to minutes before or after sunset.

*Returns to the roost—*the number of times a bat returns to the roost after the first exit, estimated as the sum of returns to the roost at each night.

*Hours inside the roost*—the total time a bat spends inside the roost, calculated as the sum of hours between each pair of consecutive coding of return–exit.

*Hours of activity*—the total time a bat spends outside the roost, calculated as the sum of time between exits and returns.

All data processing was conducted in R version 4.3.0 and Studio version 1.41103 (R Development Core Team 2023) using the package “tidyverse” (Wickham et al. 2019).

### Reproductive condition, sex, and environmental covariates

Sex (male or female) and reproductive condition of females (pregnant, lactating, postlactating, and nonreproductive) were assigned when the bats were tagged (Frick et al. 2018).

Environmental data were estimated only for the Cacachilas Complex from 2015 to 2019. We assigned food availability to each month based on the phenology of columnar cacti, the main food source for *L. yerbabuenae* in the region following the classification by Frick et al. (2018): November–January (low nectar availability); February–April (high nectar availability); May–July (high nectar–fruit availability); and August–October (high fruit availability). Daily mean temperature and precipitation were obtained from the extrapolations of the R package “daymetr” (Hufkens et al. 2018).

### Statistical analysis

We used the 4 metrics (time of emergence, frequency of returns to the roost, hours inside the roost, and hours of activity) to compare activity patterns among reproductive conditions of females using generalized linear models. We fit the metric of frequency of returns to the roost using Poisson distribution; and fit a gamma distribution with “inverse link” for hours inside the roost, time of emergence, and hours of activity, using the R package “glm” (Bolker 2021). We used the categories of lactating, pregnant, and nonreproductive as fixed effects. To test the significance of the predictors of the generalized linear models, we performed for each metric a likelihood ratio test (LRT) with a chi-squared distribution by comparing a single fixed-effect model and a null model. Then, we conducted a post hoc Tukey test for differences among reproductive conditions for the Carmen Cave data set using the function “means” from the EMMEANS package (Lenth 2022). For the alternative model with the response variable, we estimated the beta effect size of each predictor variable with 95% confidence intervals (CIs).

To determine whether bat activity patterns differ between sex and environmental factors, we fit generalized linear mixed models using the R package “glmmTMB” (Magnusson et al. 2017). We built 16 a priori interactive and additive candidate models for each activity metric. We fit the metric of frequency of returns to the roost as a negative binomial to account for overdispersion. For hours inside the roost and hours of activity, we used a gamma distribution with “log” link; and for the time of emergence, we used a Gaussian distribution (Bolker 2021). We included sex, food availability season, temperature, and precipitation as fixed effects, and ID of the bat and year as crossed random effects (Supplementary Data SD2). We used Akaike’s information criteria (AIC) for model selection (Burnham and Anderson 2002). We selected the best models based on a ΔAIC > 2 (Supplementary Data SD2). For the best-supported models, we estimated the beta effect size of each predictor variable with 95% CIs. All analyses were conducted in R version 4.3.0 and Studio version 1.41103 (R Development Core Team 2023).

## Results

### Differences in activity patterns among reproductive conditions

**Fig. 3.**
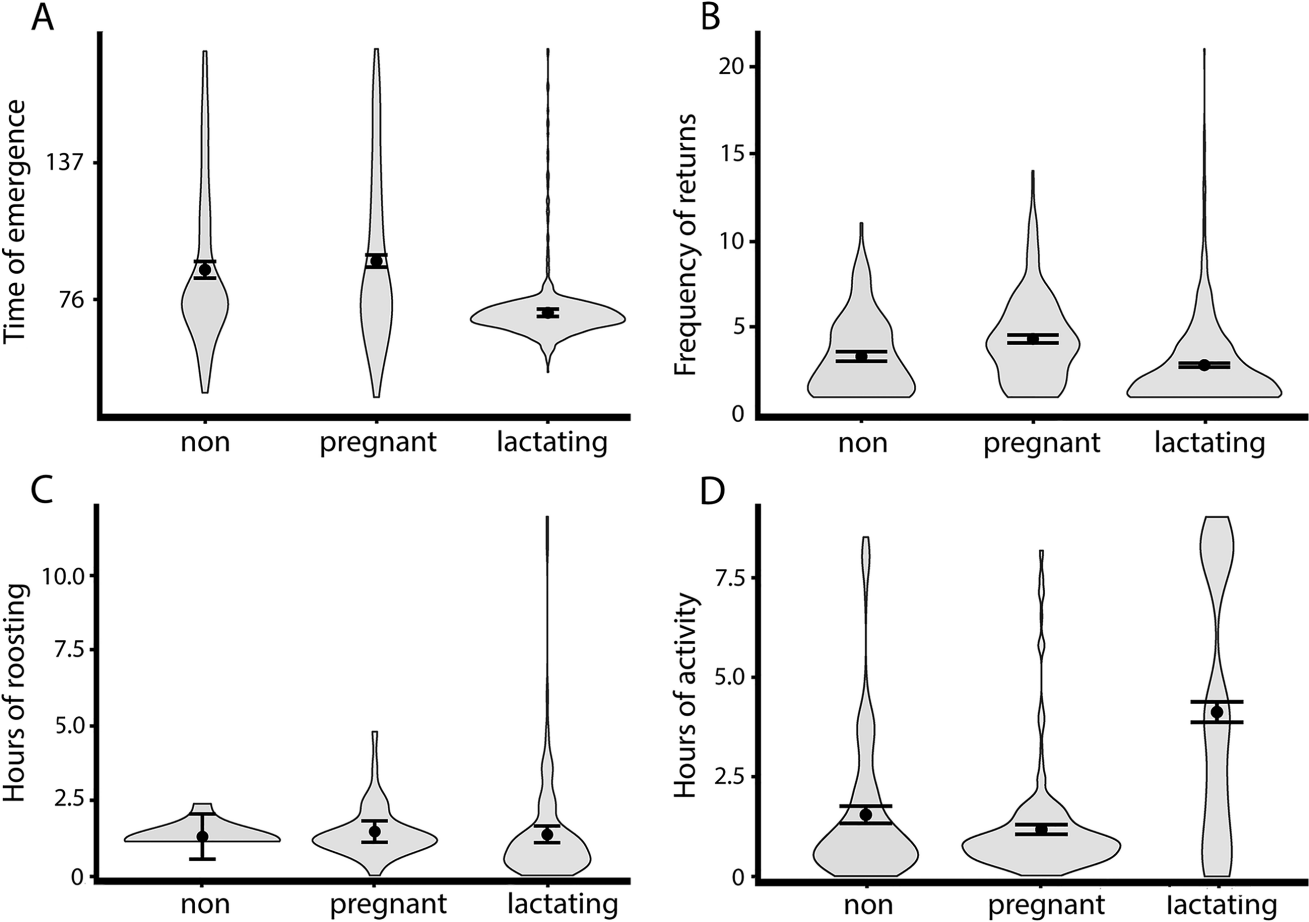
Activity patterns of *Leptonycteris yerbabuenae* for nonreproductive (non), pregnant, and lactating females. Violin plots represent the distribution of activity metrics from bat detections, points and error bars represent predicted means and 95% confidence limits from the best-supported model with the reproductive condition as fixed effect. Time of emergence is expressed as minutes after sunset. The metric of frequency of returns to the roost was fit with a Poisson distribution; while time of emergence, hours inside the roost, and hours of activity metrics were fit with gamma distribution with the “glm” function in the program R.

Activity patterns of females varied with reproductive condition when compared to a null model with a LRT for time of emergence, frequency of returns to the roost, and hours of activity outside the roost (*P* < 0.05), except for hours inside the roost (*P* = 0.97; Supplementary Data SD3). For the post hoc Tukey test, pregnant, lactating, and nonreproductive females showed significant differences in frequency of returns to the roost, hours inside the roost, and hours of activity outside the roost (*P* < 0.05; Fig. 3). Nonreproductive and pregnant females did not differ in emergence times (*P* = 0.22; Supplementary Data SD4), but lactating females emerged on average a minute earlier than pregnant females, which is not ecologically significant despite being statistically different (95% CI = 1.0006 to 1.0009, *P* < 0.001). These results confirm that female activity patterns can vary depending on reproductive condition.

Pregnant bats returned to the roost 1.47 times more each night than lactating females, which was contrary to our hypothesis (95% CI = 1.40 to 1.53, *P* < 0.001). Lactating bats had 1.27 times more hours of activity outside the roost than nonreproductive females (95% CI = 1.17 to 1.36, *P* < 0.001; Fig. 3; Supplementary Data SD4). Moreover, despite the small effect size (less than 2) of each metric, we found that lactating females are the most active outside the roost, and pregnant females returned most frequently to the roost between foraging bouts (Supplementary Data SD5).

### Sex-based and environmental differences in activity patterns

**Fig. 4.**
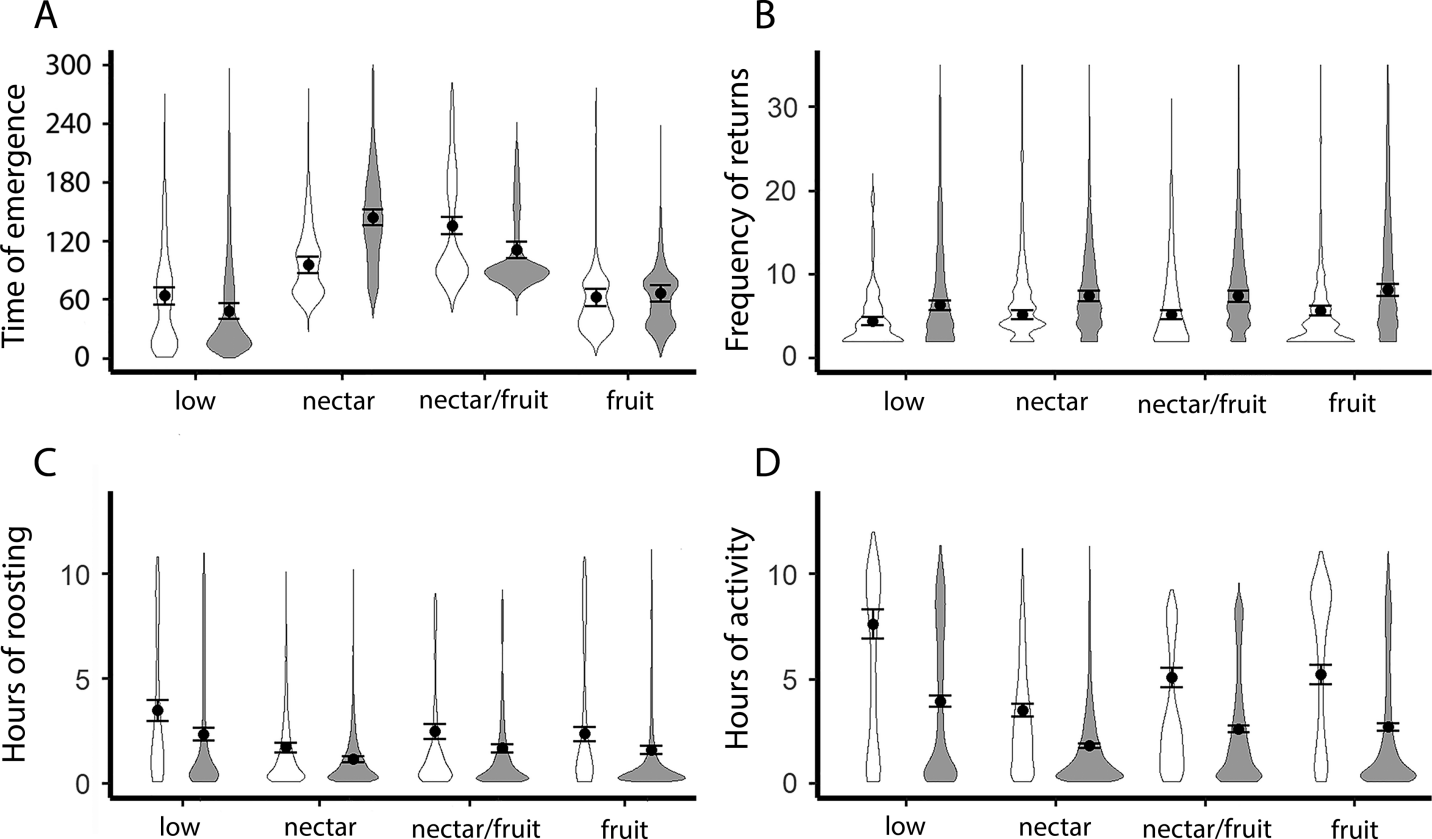
Activity patterns of *Leptonycteris yerbabuenae* for females (white) and males (gray) across 4 seasons of columnar cacti food availability in the region: low availability (November–January); nectar-only (February–April); nectar/fruit (May–July); and fruit-only (August–October). Violin plots represent the distribution of metric values; points and error bars represent predicted means and 95% confidence limits from best-supported models with sex, food availability, temperature, and precipitation as fixed effects, and ID of each bat as random effect (Supplementary Data SD6). Time of emergence is expressed as minutes after sunset. The metric of frequency of returns to the roost was fit with a Poisson distribution. While time of emergence, hours inside the roost, and hours of activity metrics were fit with gamma distribution with the “glmmTMB” package in the program R. We used mean precipitation and temperature values for model predictions. See Supplementary Data SD6 for more details.

On average, males returned to the roost 1.43 times more per night (95% CI = 1.28 to 1.60, *P* < 0.05), emerged 15 min before females (95% CI = 10.90 to 20.3, *P* < 0.05), and spent 40 min less inside the roost than females (95% CI = 39.88 to 40.11, *P* < 0.05). On average, males were 4.95 h less active outside the roost than females (95% CI = 4.85 to 5.04, *P* < 0.001; Fig. 4; Supplementary Data SD6).

We found that activity patterns of males and females varied according to environmental factors such as food availability and precipitation (Supplementary Data SD6). The time of emergence for males during low food availability was 75 min earlier than females (95% CI = 74.87 to 75.127, *P* < 0.001). However, during the nectar season which overlaps with the female reproductive season, males emerged 90 min later than females (95% CI = 87 to 98, *P* < 0.001; Fig. 4). For models using time of emergence as the response variable, there was a significant interaction term between sex and food availability (Supplementary Data SD2).

For frequency of returns, males returned 2.24 to 2.5 times more than females during the nectar (95% CI = 2.1 to 2.3, *P* < 0.001) and low food availability season (95% CI = 2.4 to 2.55, *P* < 0.001; Fig. 4). For hours inside the roost, males stayed 48 min less time than females during the low food availability season (95% CI = 47 to 49, *P* < 0.001, Fig. 4). For hours of activity, males were 3.7 h less active outside the roost than females during the low food availability (95% CI = 3.6 to 3.7, *P* < 0.001), and 1.7 h less active outside the roost in nectar season (95% CI = 1.06 to 1.72, *P* < 0.001; Fig. 4). Models that included precipitation and temperature as covariates had the best fit in all the models, except in the best-fit model for the response of hours of activity outside the roost that does not include temperature (Supplementary Data SD6).

## Discussion

Nightly activity patterns of *L. yerbabuenae*, a colonially roosting nectar-feeding bat, varied with reproductive status, sex, and food availability. As expected, females spent more time active outside the roost than males, while lactating females spent significantly more time active outside the roost than pregnant or nonreproductive females. Contrary to our expectations, lactating females returned to the roost less frequently during the night, suggesting that lactating females extend rather than interrupt their foraging bouts to return to nurse and care for pups.

We predicted that lactating females would return more often to the roost each night to nurse pups (Kleiman 1969; Ripperger et al., 2019), but we found the opposite pattern. This was probably because lactating females of *L. yerbabuenae* form maternity colonies in “hot roosts” with sufficiently high internal roost temperatures, and they strategically place their pups in large nursery groups, increasing the temperature in the core of the nursery (Ávila-Flores and Medellín 2004; Iñarritu Castro 2017; Goldshtein et al. 2020). These 2 features may help pups thermoregulate while mothers are foraging and reduce dependency of the pup on the mother.

Some lactating bats stayed for long extended periods inside the roost (some individuals stayed for 5 to 12 h inside), which is consistent with some females engaging in crèche behavior caring for young while roost-mates forage, which has been observed with other colonially roosting species such as *Tadarida brasiliensis* (McCracken and Gustin 1991) and species of the Phyllostomidae family such as *Phyllostomus hastatus* (Wilkinson et al. 2016).

Our results are consistent with other studies showing that female bats adjust their foraging strategies to meet the energetic demands of lactation. Lactation is energetically demanding as females must invest energy to care for and feed their pups and produce milk (Speakman 2008), resulting in a continuous reduction of their energetic reserves (Fleming et al. 1998; Martínez-Coronel et al. 2014). For example, in insectivorous bats, the food consumption of lactating females can increase up to 1.5 to 6 times compared to nonreproductive females (McLean and Speakman 1999).

*Leptonycteris yerbabuenae* lactating females in cacti-dense ecosystems are income breeders because they mostly allocate energy obtained from external nutrients during the same season to subsidize milk production as their body reserves are insufficient (Ramírez Hernández and Herrera 2016). To compensate for these energetic demands, lactating females may require longer foraging bouts (Jones and Rydell 1994; Kunz et al. 1995; McLean and Speakman 1999; Duvergé et al. 2000; Goldshtein et al. 2020). Water intake needs could be a complementary reason for the difference in activity patterns, especially in lactating females. In some insectivorous species, bat milk constitutes nearly 76% water (Adams and Hayes 2021), and lactating females can drink water 7 times more than nonreproductive females (Adams and Hayes 2008). However, more research is needed to understand whether water intake needs in nectarivorous bats affect their activity patterns as it is suggested for other species.

Pregnancy is also an energetically demanding condition (Speakman 2008) that requires being active outside the roost foraging (Charles-Dominique 1991). However, pregnant bats have a trade-off between the amount of time they need to forage to meet energetic demands, and reduced mobility caused by the weight of the embryo, which can grow up to 40% of the weight of the mother before parturition (Duvergé et al. 2000; Frick et al 2018). We expected that pregnant individuals would stay the longest inside the roost and show the shortest activity, as has been observed with the frugivorous bat *Carollia perspicillata* (Charles-Dominique 1991). While this was generally true, some pregnant females in our study had long hours of activity outside the roost (up to 7 h), which could have occurred during early pregnancy (Rydell 1993). Pregnant females returned more frequently, perhaps due to reduced maneuverability during gestation. Females arrive at Carmen Cave roost in pregnant conditions in spring, often before the peak bloom of columnar cacti (highest nectar availability; Frick et al. 2018). Nectar availability may have a strong influence on the time female bats spend outside the roost, but we cannot disentangle that from reproductive condition given the overlap in timing.

Females consistently spent more time active outside the roost than males in all seasons. Longer foraging times for females have been observed in other bat species, such as *Triaenops furculus* (Olsson et al. 2006) probably because of energetic demands, but not in others such as *Macrophyllum macrophyllum* and *C. castanea* where there are no differences recorded between sexes, probably because the nonreproductive males and females analyzed do not have high energetic demands differences (Thies et al. 2006; Weinbeer et al. 2006). In our study, male *L. yerbabuenae* returned more frequently to the roost than females, especially during the fruit availability season, which coincides with their mating season. At this time, males are likely to focus their activity inside the roost to court females (Stoner et al. 2003; Encarnação et al. 2004). Studies on other bats found that male bats can return a few times to the roost during the night (Thies et al. 2006). However, most of the behavioral studies of bat activity have focused on females, resulting in a more limited understanding of activity patterns for males (Hałat et al. 2018).

Timing of emergence did not vary substantially among females with different reproductive conditions, which contrasts with patterns observed for some insectivorous bat species such as *Eptesicus nilssonii*, *Rhinolophus ferrumequinum* (Duvergé et al. 2000), and *Lasiurus cinereus* (Barclay 1989). In insectivorous bats, the timing of emergence can be influenced by climatic conditions and food availability (Frick et al 2012; Thomas and Jacobs 2013). In our study, the main food available for bats during the nectar season is from the columnar cactus *Pachycereus pringlei* (April–May; Frick et al. 2018). Flowers open shortly after sunset, but the peak in their nectar production is in the middle of the night (Fleming et al. 1994). During the nectar season, which corresponds with maternity season, females emerged significantly earlier than males, a behavior similar to other bat species (Shiel and Fairley 1999). Patterns in the timing of emergence may have different fitness consequences for nectarivorous bats than insectivorous species (Frick et al. 2012; Thomas and Jacobs 2013) if food abundance peaks later in the night. Some physiological traits might be influencing the activity patterns found here as previous studies in laboratory trials have found that *L. yerbabuenae* bats increase their feeding time when sugar concentration in nectar is low; while they reduce their food intake when concentration increases (Ayala-Berdon et al. 2011). Thus, it is worth exploring whether activity patterns of bats adapt hourly according to the production of nectar of their preferred and most abundant food sources.

### Final remarks

Our activity metrics were calculated from passively monitored PIT-tag detections in the entrance of 3 subterranean roosts of *L. yerbabuenae*, a migratory nectar-feeding bat. While there are some limitations to inferring exactly how bats spent their time inside and outside the roost, these data provided a new way to assess activity patterns of a colonially roosting bat species. Long-term maintenance of RFID readers at roost entrances and protection of known roosts can aid in monitoring of population status and activity patterns of *L. yerbabuenae* across their range (Fontaine et al. 2024). As more RFID readers and roost entrance antennae are installed across the range of *L. yerbabuenae*, the more we can understand variation in activity patterns across space and time in relation to climate and land use change to help inform species conservation efforts.

## Supplementary data

Supplementary data are available at *Journal of Mammalogy* online.

**Supplementary Data SD1.** Deriving activity metrics from PIT-tag detections.

**Supplementary Data SD2.** A priori hypotheses of the interactive and additive generalized linear mixed models on how activity patterns vary according to sex and environmental conditions. We show the model structure, degrees of freedom, and ΔAIC of each model at the time of emergence, frequency of returns to the roost, time spent inside the roost, and hours outside the roost as response variables; we included sex, food availability, temperature, and precipitation as fixed effects, and ID of the bat and year as crossed random effects. The best-supported models are the ones with a ΔAIC > 2.

**Supplementary Data SD3.** Likelihood ratio test (LRT) of the response variables of the generalized linear models on how activity patterns vary according to the life-history traits of *Leptonycteris yerbabuenae* females. We compare the single fixed-effect models (reproductive conditions) with null models with only intercept.

**Supplementary Data SD4.** Multiple comparisons of post hoc for the generalized linear model according to females’ reproductive condition. Multiple comparisons of post hoc for the generalized linear model for each metric of the activity patterns of female bats of *Leptonycteris yerbabuenae* according to their reproductive condition. This shows that all reproductive conditions influence the activity patterns differently from each other.

**Supplementary Data SD5.** Coefficient estimates of the 4 metrics of activity patterns for female bats of *Leptonycteris yerbabuenae* according to their reproductive condition. Generalized linear models of each of the 4 metrics of activity patterns for female bats of *L. yerbabuenae* according to their reproductive condition. The intercept is the pregnant females.

**Supplementary Data SD6.** Results of best-supported models of life-history traits and environmental factors that influence activity patterns. Only ΔAIC < 2 are shown. The time of emergence was measured in hours relative to sunset. The frequency of returns to the roost is the sum of roost entries in a night. Hours inside the roost is the sum of time between each pair of entries and exits between sunset and sunrise, reflecting the period that bats are active. Hours of activity is the sum of time between exits and entrances between sunset and sunrise. The estimates recorded for time of emergence are from the interactive model, while for frequency of returns, hours inside the roost, and hours of activity are their best-supported additive models. Female and high fruit seasons were used as the reference group.

gyae092_suppl_Supplementary_Data_SD1

gyae092_suppl_Supplementary_Data_SD2

gyae092_suppl_Supplementary_Data_SD3

gyae092_suppl_Supplementary_Data_SD4

gyae092_suppl_Supplementary_Data_SD5

gyae092_suppl_Supplementary_Data_SD6

## Data Availability

R scripts and clean daily detections are available at GitHub: https://github.com/VeroZamora/Activity-patterns-Leptonycteris-yerbabuenae.git.
