# Supplementary material for: Activity patterns of the nectar-feeding bat Leptonycteris yerbabuenae on the Baja California Peninsula, Mexico
Source: J Mammal. 2024 Aug 19;105(6):1221–30. doi: 10.1093/jmammal/gyae092 (PMC11586102; doi:10.1093/jmammal/gyae092)
Supplement: gyae092_suppl_Supplementary_Data_SD1 [file gyae092_suppl_supplementary_data_sd1.docx]

**Supplementary Data SD1.**— Deriving activity metrics from PIT-tag detections.

We processed tag detections under certain rules to derive four metrics of bat activity: time of emergence, returns to the roost, hours inside the roost (hours roosting), and hours outside the roost (hours of activity). We addressed three potential sources of error in the data. (1) Missed detections: when a bat enters or exits a roost, but the PIT-tag antenna fails to record it because the bat flew outside the range of detection of the antenna. (2) Possible roost movements: when a bat only records one PIT-tag detection per night that is either an enter or an exit, it could reflect a roost movement because there are other roosts available in the area. To deal with missed detections and possible roost movements, we excluded the data of those bats that were only detected once a night on the presumption that a bat should have at least two detections in a night (one for the exit and one for the return to the roost). (3) Non-exit detections: a bat flies near the entrance within the range of detection by the antenna but does not leave the roost. To reduce this error, we consider the time of emergence for the night, the time when the first daily 75% PIT-tag detections of the bats were recorded. Once the detection was labeled as emergence (first exit of the night), we assigned the subsequent detections as entry or exit by the following criteria: 1) we labeled only those detections that were at least 15 minutes apart to include the most plausible detections of true exit and return activity; 2) we included only the detections recorded from sunset to sunrise.
