# Supplementary material for: Activity patterns of the nectar-feeding bat Leptonycteris yerbabuenae on the Baja California Peninsula, Mexico
Source: J Mammal. 2024 Aug 19;105(6):1221–30. doi: 10.1093/jmammal/gyae092 (PMC11586102; doi:10.1093/jmammal/gyae092)
Supplement: gyae092_suppl_Supplementary_Data_SD2 [file gyae092_suppl_supplementary_data_sd2.docx]

**Supplementary Data SD2.**— *A priori* hypotheses of the interactive and additive Generalized Linear Mixed Models on how activity patterns vary according to sex and environmental conditions. We show the model structure, degrees of freedom, and ΔAIC of each model at the time of emergence, frequency of returns to the roost, time spent inside the roost, and hours outside the roost as response variables; we included sex, food availability, temperature, and precipitation as fixed effects, and ID of the bat and year as crossed random effects. The best-supported models are the ones with a ΔAIC > 2. Food = food availability season, precip = precipitation, temp= temperature. The interactive models are identified with the asterisk (*) sign.

| **Response variable** | **Family distribution** | **Model** | **df** | **dAIC** |
| --- | --- | --- | --- | --- |
| Time of emergence | Gaussian | sex * food + temp + precip + (1\|id) + (1\|year) | 13 | 0 |
|  |  | sex + food + temp + precip + (1\|id) + (1\|year) | 10 | 6689.69 |
|  |  | food + temp + precip + (1\|id) + (1\|year) | 9 | 6735.21 |
|  |  | sex + food + temp + (1\|id) + (1\|year) | 9 | 6816.68 |
|  |  | food + temp + (1\|id) + (1\|year) | 8 | 6862.24 |
|  |  | sex + food + precip + (1\|id) + (1\|year) | 9 | 7132.52 |
|  |  | food + precip + (1\|id) + (1\|year) | 8 | 7176.34 |
|  |  | sex + food +(1\|id) + (1\|year) | 8 | 7251.13 |
|  |  | food + (1\|id) + (1\|year) | 7 | 7295.02 |
|  |  | temp + precip + (1\|id) + (1\|year) | 6 | 66204.84 |
|  |  | sex + temp + (1\|id) + (1\|year) | 6 | 66860.68 |
|  |  | temp +(1\|id) + (1\|year) | 5 | 66869.34 |
|  |  | sex + precip + (1\|id) + (1\|year) | 6 | 69844.95 |
|  |  | precip + (1\|id) + (1\|year) | 5 | 69847.64 |
|  |  | sex + (1\|id) + (1\|year) | 5 | 70548.64 |
|  |  | (1\|id) + (1\|year) | 4 | 70551.27 |
| Frequency of returns to the roost | Negative Binomial | sex + food + temp + precip + (1\|id) + (1\|year) | 10 | 0 |
|  |  | sex + food + temp + (1\|id) + (1\|year) | 9 | 3.96 |
|  |  | food + temp + precip + (1\|id) + (1\|year) | 9 | 38.09 |
|  |  | food + temp + (1\|id) + (1\|year) | 8 | 42.05 |
|  |  | sex + food + precip + (1\|id) + (1\|year) | 9 | 386.92 |
|  |  | sex + food +(1\|id) + (1\|year) | 8 | 388.68 |
|  |  | food + precip + (1\|id) + (1\|year) | 8 | 425.56 |
|  |  | food + (1\|id) + (1\|year) | 7 | 427.32 |
|  |  | sex + temp + (1\|id) + (1\|year) | 6 | 1314.31 |
|  |  | temp + precip + (1\|id) + (1\|year) | 6 | 1334.22 |
|  |  | temp +(1\|id) + (1\|year) | 6 | 1349.57 |
|  |  | sex + precip + (1\|id) + (1\|year) | 6 | 3534.51 |
|  |  | sex + (1\|id) + (1\|year | 5 | 3553.39 |
|  |  | precip + (1\|id) + (1\|year) | 5 | 3576.24 |
|  |  | (1\|id) + (1\|year) | 4 | 3595.16 |
| Hours inside the roost | Gamma | sex + food + temp + precip + (1\|id) + (1\|year) | 10 | 0 |
|  |  | sex + food + temp + (1\|id) + (1\|year) | 9 | 15.32 |
|  |  | food + temp + precip + (1\|id) + (1\|year) | 9 | 43.95 |
|  |  | food + temp + (1\|id) + (1\|year) | 8 | 59.13 |
|  |  | sex + food + precip + (1\|id) + (1\|year) | 9 | 323.64 |
|  |  | sex + food +(1\|id) + (1\|year) | 8 | 340.38 |
|  |  | food + precip + (1\|id) + (1\|year) | 8 | 367.65 |
|  |  | food + (1\|id) + (1\|year) | 7 | 384.25 |
|  |  | temp + precip + (1\|id) + (1\|year) | 6 | 1657.55 |
|  |  | sex + temp + (1\|id) + (1\|year) | 6 | 1659.3 |
|  |  | temp +(1\|id) + (1\|year) | 5 | 1678.83 |
|  |  | sex + precip + (1\|id) + (1\|year) | 6 | 2232.6 |
|  |  | sex + (1\|id) + (1\|year) | 5 | 2249.41 |
|  |  | precip + (1\|id) + (1\|year) | 5 | 2259.79 |
|  |  | (1\|id) + (1\|year) | 4 | 2276.37 |
| Hours of activity | Gamma | sex + food + precip + (1\|id) + (1\|year) | 9 | 0 |
|  |  | sex + precip + (1\|id) + (1\|year) | 10 | 1.96 |
|  |  | sex + food + temp + precip + (1\|id) + (1\|year) | 10 | 1.96 |
|  |  | sex + food +(1\|id) + (1\|year) | 8 | 2.63 |
|  |  | sex + temp + (1\|id) + (1\|year) | 8 | 2.63 |
|  |  | sex + food + temp + (1\|id) + (1\|year) | 9 | 4.6 |
|  |  | food + precip + (1\|id) + (1\|year) | 8 | 130.62 |
|  |  | precip + (1\|id) + (1\|year) | 8 | 130.62 |
|  |  | food + temp + precip + (1\|id) + (1\|year) | 9 | 132.6 |
|  |  | food + (1\|id) + (1\|year) | 7 | 133.3 |
|  |  | food + temp + (1\|id) + (1\|year) | 8 | 135.29 |
|  |  | sex + (1\|id) + (1\|year) | 5 | 3351.71 |
|  |  | temp + precip + (1\|id) + (1\|year) | 6 | 3432.04 |
|  |  | (1\|id) + (1\|year) | 4 | 3437.87 |
|  |  | temp +(1\|id) + (1\|year) | 5 | 3439.11 |
