# Supplementary material for: Activity patterns of the nectar-feeding bat Leptonycteris yerbabuenae on the Baja California Peninsula, Mexico
Source: J Mammal. 2024 Aug 19;105(6):1221–30. doi: 10.1093/jmammal/gyae092 (PMC11586102; doi:10.1093/jmammal/gyae092)
Supplement: gyae092_suppl_Supplementary_Data_SD3 [file gyae092_suppl_supplementary_data_sd3.docx]

**Supplementary Data SD3.**— Likelihood ratio test (LRT) of the response variables of the generalized linear models on how activity patterns vary according to the life-history traits of *L. yerbabuenae* females. We compare the single fixed effect models (reproductive conditions) with null models with only intercept.

| **Response variable** | **Distribution** | **LRT** | **Degrees of freedom** | **p-value** |
| --- | --- | --- | --- | --- |
| Time of emergence | Gamma | 231.1962 | 4 | **0.00** |
| Frequency of returns | Poisson | 152.5042 | 3 | **0.00** |
| Hours inside the roost | Gamma | 0.4561813 | 4 | 0.9776241 |
| Hours of activity | Gamma | 313.7415 | 4 | **0.00** |
