# Supplementary material for: Activity patterns of the nectar-feeding bat Leptonycteris yerbabuenae on the Baja California Peninsula, Mexico
Source: J Mammal. 2024 Aug 19;105(6):1221–30. doi: 10.1093/jmammal/gyae092 (PMC11586102; doi:10.1093/jmammal/gyae092)
Supplement: gyae092_suppl_Supplementary_Data_SD4 [file gyae092_suppl_supplementary_data_sd4.docx]

**Supplementary Data SD4.**— Multiple comparisons of post-hoc for the generalized linear model according to females' reproductive condition.

Multiple comparisons of post-hoc for the generalized linear model for each metric of the activity patterns of female bats of *Leptonycteris yerbabuenae* according to their reproductive condition. This shows that all reproductive conditions influence the activity patterns differently from each other.

| **Metric** | **Conditions** | **Estimate** | **SE** | **Z ratio** | **p-value** |
| --- | --- | --- | --- | --- | --- |
| Time of emergence | Lactating – non-reproductive | 0.000791 | 8.48e^-05^ | 9.325 | <0.0001 |
|  | Lactating – pregnant | 0.000948 | 6.58e^-05^ | 14.404 | <0.0001 |
|  | Non-reproductive – pregnant | 0.000157 | 9.54e^-05^ | 1.650 | 0.2251 |
| Frequency of returns to the roost | Lactating – non-reproductive | -0.161 | 0.0470 | -3.427 | 0.0018 |
|  | Lactating – pregnant | -0.425 | 0.0337 | -12.615 | <.0001 |
|  | Non-reproductive - pregnant | -0.264 | 0.0501 | -5.271 | <.0001 |
| Hours inside the roost | Lactating – non-reproductive | -1.51e^-05^ | 2.63e^-06^ | -5.738 | <.0001 |
|  | Lactating – pregnant | -7.65e^-06^ | 1.82e^-06^ | -4.205 | 0.0001 |
|  | Non-reproductive - pregnant | 7.42e-06 | 2.91e^-06^ | 2.551 | 0.0294 |
| Hours of activity | Lactating – non- reproductive | -0.402 | 0.0466 | -8.628 | <.0001 |
|  | Lactating – pregnant | -0.604 | 0.0444 | -13.596 | <.0001 |
|  | Non-reproductive - pregnant | -0.202 | 0.0635 | -3.178 | 0.0043 |
