# Supplementary material for: Activity patterns of the nectar-feeding bat Leptonycteris yerbabuenae on the Baja California Peninsula, Mexico
Source: J Mammal. 2024 Aug 19;105(6):1221–30. doi: 10.1093/jmammal/gyae092 (PMC11586102; doi:10.1093/jmammal/gyae092)
Supplement: gyae092_suppl_Supplementary_Data_SD5 [file gyae092_suppl_supplementary_data_sd5.docx]

**Supplementary Data SD5.**— Coefficient estimates of the four metrics of activity patterns for female bats of *Leptonycteris yerbabuenae* according to their reproductive condition.

Generalized Linear Models of each of the four metrics of activity patterns for female bats of *L. yerbabuenae* according to their reproductive condition. The intercept is the pregnant females.

| **Metric** | **Conditions** | **Estimate** | **SE** | **t value** | **p-value** |
| --- | --- | --- | --- | --- | --- |
| Time of emergence | Intercept | 0.04947 | 3.483e^-05^ | 1442.96 | < 0.0001 |
|  | Non-reproductive | -0.00079 | 9.536e^-05^ | -1.650 | 0.0992 |
|  | Lactating | 0.00079 | 8.48e^-05^ | 9.325 | < 0.0001 |
| Frequency of returns to the roost | Intercept | 1.20637 | 0.020 | 51.37 | < 0.0001 |
|  | Non-reproductive | 0.16097 | 0.047 | 3.42 | 0.0006 |
|  | Pregnant | 0.42 | 0.033 | 12.61 | < 0.0001 |
| Hours inside the roost | Intercept | 0.8415 | 0.2532 | 3.323 | 0.00105 |
|  | Pregnant | -0.1590 | 0.2666 | -0.596 | 0.55 |
|  | Lactating | -0.1003 | 0.2624 | -0.382 | 0.70267 |
| Hours of activity | Intercept | 0.64457 | 0.04598 | 14.017 | < 0.0001 |
|  | Pregnant | 0.20173 | 0.06347 | 3.178 | 0.00152 |
|  | Lactating | -0.40222 | 0.04662 | -8.628 | < 0.0001 |
