# Supplementary material for: Activity patterns of the nectar-feeding bat Leptonycteris yerbabuenae on the Baja California Peninsula, Mexico
Source: J Mammal. 2024 Aug 19;105(6):1221–30. doi: 10.1093/jmammal/gyae092 (PMC11586102; doi:10.1093/jmammal/gyae092)
Supplement: gyae092_suppl_Supplementary_Data_SD6 [file gyae092_suppl_supplementary_data_sd6.docx]

**Supplementary Data SD6.**— Results of best-supported models of life history traits and environmental factors that influence activity patterns. Only (ΔAIC < 2) are shown. The time of emergence was measured in hours relative to the sunset. The frequency of returns to the roost is the sum of roost entries in a night. Hours inside the roost is the sum of time between each pair of entries and exits between the sunset and sunrise, reflecting the period that bats are active. Hours of activity is the sum of time between exits and entrances between the sunset and sunrise. The estimates recorded for time of emergence are from the interactive model, while for frequency of returns, hours inside the roost, and hours of activity are their best-supported additive models. Female and high fruit seasons were used as the reference group.

| **Response** | **Variable** | **Estimate** | **SE** | ***Z*-value** | ***p-*value** |
| --- | --- | --- | --- | --- | --- |
| **Time of emergence** | Intercept | 1.43 | 0.077 | 18.62 | < 0.0001 |
|  | Sex (male) | -0.26 | 0.04 | -6.43 | < 0.0001 |
|  | Food Availability (nectar) | 0.53 | 0.02 | 23.26 | < 0.0001 |
|  | Food Availability (nectar and fruit) | 1.19 | 0.02 | 48.73 | < 0.0001 |
|  | Food Availability (Low) | -0.02 | 0.025 | -1.12 | < 0.0001 |
|  | Precipitation | -0.004 | 0.00038 | -11.49 | < 0.0001 |
|  | Temperature | -0.014 | 0.0006 | -21.15 | < 0.0001 |
| **Frequency of returns to the roost** | Intercept | 1.40 | 0.058 | 24 | < 0.0001 |
|  | Sex (male) | 0.36 | 0.055 | 6.5 | < 0.0001 |
|  | Food Availability (nectar) | -0.087 | 0.0066 | -13 | < 0.0001 |
|  | Food Availability (nectar and fruit) | -0.095 | 0.0045 | -21 | < 0.0001 |
|  | Food Availability (low) | -0.25 | 0.0075 | -33 | < 0.0001 |
|  | Temperature | 0.014 | 0.00071 | 20 | < 0.0001 |
|  | Precipitation | 0.00098 | 0.0004 | 2.4 | < 0.0001 |
| **Hours inside the roost** | Intercept | 1.8 | 0.09 | 19 | < 0.0001 |
|  | Sex (male) | -0.39 | 0.057 | -7 | < 0.0001 |
|  | Food Availability (nectar) | -0.32 | 0.017 | -18 | < 0.0001 |
|  | Food Availability (nectar and fruit) | 0.0540 | 0.012 | 4.6 | < 0.0001 |
|  | Food Availability (Low) | 0.3900 | 0.0200 | 20.0 | < 0.0001 |
|  | Precipitation | 0.0043 | 0.0011 | 4 | < 0.0001 |
|  | Temperature | -0.035 | 0.0019 | -18 | < 0.0001 |
| **Hours of activity** | Intercept | 1.70 | 0.046 | 36.0 | < 0.0001 |
|  | Sex (male) | -0.66 | 0.051 | -13 | < 0.0001 |
|  | Precipitation | 0.0018 | 0.00084 | 2.1 | < 0.0001 |
|  | Food Availability (nectar) | -0.40 | 0.011 | -36 | < 0.0001 |
|  | Food Availability (nectar and fruit) | -0.029 | 0.0093 | -3.2 | < 0.0001 |
|  | Food Availability (Low) | 0.38 | 0.012 | 31 | < 0.0001 |
